# Study on the time and scale of mutual aid for aging care under the background of active aging

**DOI:** 10.3389/fpubh.2023.1196411

**Published:** 2024-01-15

**Authors:** Wenguang Yu, Qi Wang, Zhi Qiao

**Affiliations:** ^1^School of Insurance, Shandong University of Finance and Economics, Jinan, China; ^2^School of Statistics and Mathematics, Shandong University of Finance and Economics, Jinan, China; ^3^School of Insurance, Central University of Finance and Economics, Beijing, China

**Keywords:** mutual aid, aging care, time for care, the size of the care group, overlapping generations model

## Abstract

**Background:**

China has entered an aging society and will likely become the fastest-aging country in the world. The demand for aging care services has greatly increased. In recent years, the model of mutual aid for aging care has met the needs of older adults, especially those in rural areas. In this case, how much time should be spent on mutual aid for the older adult in terms of time and how much influence the size of the group has in terms of space are two very important questions when studying mutual aid for aging care.

**Methods:**

An overlapping generations model is built in this article, which includes representative agents, representative enterprises, the endowment insurance system constructed by the government, and the behavior of representative agents in mutual aid for aging care under the background of active aging.

**Results:**

In the base case, the optimal proportion of time to participate in the mutual aid group is 9.31%, and the optimal proportion of time is influenced by the benchmark time of care and the size of the care group. With the increase in the benchmark time of care, the optimal proportion of time increases correspondingly, but the increase is decreasing. With the increase in the size of the care group, the optimal proportion of time decreases, but after the size reaches 4 or 5, the impact becomes very small. When parents' psychological preference coefficient changes from 0.1 to 0.9, that is, when parents change from introverted to extroverted personalities, the optimal proportion of time and parents' utility will also change.

**Conclusion:**

For children who usually take care of their parents for a long benchmark time, the optimal time to participate in the mutual aid group based on personal utility maximization is also long. Second, as the size of the group increases, the time for representative agents to participate in the mutual aid group gradually decreases. In addition, the model of mutual aid for aging care is highly correlated with parents' personalities, and extrovert parents benefit more from this model.

## Introduction

There were 267 million people aged 60 or older in China at the end of 2021, or 18.9% of the total population.^1^ China has entered an aging society and will likely become the fastest-aging country in the world. In this context, the demand for aging care services has greatly increased. In recent years, aging care modes such as neighborhood mutual aid courtyards, age-friendly communities, and community day-care centers have gradually emerged in various places. With the aggravation of the aging problem, the life care, medical care, cultural education, and spiritual comfort of the older adult have become important issues for them. The proportion of the older adult is rising, and young people are flowing to the cities, resulting in a more serious problem of aging care in rural China. The traditional way of relying on family for aging care is weakening. In recent years, the model of mutual aid for aging care has met the needs of the older adult, especially those in rural areas, and this model has also received the attention of the national civil affairs department. For example, in Shaanxi Province, more than two older adults spontaneously form a group, and the members are relatives, friends, or neighbors. They have known each other for a long time and are familiar with each other's personalities, so they can form a stable relationship. This group has 1–2 families at least and 4–5 families at most.^2^ They either rent a house or use the house of one of the members to take care of each other in life. In this mode, their children no longer rely on themselves to take care of their parents but take turns to provide care for these older adults. This method not only reduces the time for children to take care of older adults but also improves their happiness. Therefore, this model is not only recognized by older adults in rural area, but many urban older adults also go to rural areas for mutual support.

Living in a rapidly aging country, older adults in China pay more attention to these issues of aging. Using the data from the Chinese Longitudinal Healthy Longevity Survey, Zhang et al. analyzed the accessibility of healthcare and find that the perception of the older adult on medical care in China increased from 89.6 to 96.7% (1). In terms of medical and non-medical services for the older adult, Di et al. evaluated the feasibility of home-based medical services for the older adult (2), while Yu et al. analyzed the related factors of non-medical services (3). The problems faced by the older adult in medical and non-medical services are summarized as insufficient information and economic burden by Atakro et al. (4).

For the sources of care, Boerner et al. analyzed the sources and types of support in the daily lives of the older adult. The results show that children are the primary source of support for the older adult in daily life. Therefore, the older adult without children received less help in general, and they are more likely to ask friends or neighbors to help them with housework (5). Sudo et al. analyzed four kinds of aging care policies in Japan and pointed out that Japan's community-based integrated care system mainly has four key factors: self-help, mutual aid, social solidarity care, and government care (6). These measures can provide experience for other aging countries. Hossain et al. revealed the differences in gender, marriage, and other characteristics in the use of gerontological services (7). In the context of filial culture, Zang pointed out that when Chinese senior people choose the type of care, the influence of filial culture is very important (8).

Mutual assistance for senior citizens can help them enjoy life better, but whether they can successfully implement mutual assistance depends on their willingness and acceptance. Yao et al. evaluated the needs of the older adult in rural China for mutual help and found that senior citizens in rural areas have a high demand for mutual help. Promoting mutual help is conducive to improving the happiness of senior citizens (9). Long-term care institutions are one of the most common ways to provide care services and achieve mutual help for the older adult at this stage. Huang et al. analyzed the influencing factors of whether older adults are willing to enter long-term care institutions, and the results show that only 11.9% of the respondents were willing to enter long-term care facilities to meet their medical needs. When choosing long-term care institutions, most people are also more willing to enter those institutions close to their families (10). Older adults represent the largest consumers of health; through a cross-sectional study of 117 clients in an aging care facility in Australia, Hobden et al. pointed out that senior people did not receive adequate patient-centered care in the care facility (11). The empty-nest senior citizens receive less care, and Qian et al. analyzed the willingness of these groups for institutional care and found that empty-nest senior citizens are more willing to accept care from institutions (12). Comparing home-based care with institutional care, Wang et al. found that family eldercare is the priority for older adults compared with community-based and institutional eldercare (13).

Social support and mutual help not only solve various aging problems faced by the older adult but also have an inevitable impact on their health and wellbeing. Kim and Lee analyzed the impact of social support on the health of the older adult and pointed out that social support can improve the quality of a healthy life (14). Bai et al. analyzed the impact of social support on the older adult in rural China and pointed out that telephone communication with children and accompanying grandchildren can significantly improve the health of the older adult in rural areas. It is necessary for young people to provide emotional support to promote the health of the older adult (15). Using the data from the China Longitudinal Aging Social Survey, Li et al. also proved that intergenerational relationships and family social support can significantly reduce depression among Chinese senior citizens (16). In addition to the impact on health, social support and mutual assistance for older adults can also affect the wellbeing of senior citizens. Ryu and Heo found that participation in volunteer activities can help improve the happiness of old adults (17). Nanbu et al. analyzed the impact of mutual assistance among rural residents on psychological pressure, and the result indicated that self-help and mutual assistance among senior citizens play an important role (18). Murayama et al. analyzed the mental health of the older adult in different situations such as family mutual aid, neighborhood mutual aid, and no mutual aid, and the result showed that mutual aid can effectively improve the mental health of the older adult (19). These studies confirm that social support and mutual help have a positive effect on the health and happiness of the older adult.

In terms of the model of mutual aid for the older adult, different countries have formed a variety of models according to their characteristics. Greenfield et al. analyzed the concept and key issues of the American Age-Friendly Community Initiatives (AFCI) and pointed out that AFCI can involve stakeholders from multiple departments, which is conducive to the health and wellbeing of the older adult (20). Xue and Chul analyzed the different mutual aid models in various countries such as the Capitol Hill Village in the United States, the Multi-generation House in Germany, the Suzuki Club in Japan, and the Baba Yajia in France. In addition, they also analyzed several models developed in the process of exploring mutual aid for the older adult in China such as the Time Bank in Jiangyan, the Xingfu Home in Feixiang, the Stronghold Activities in Qingdao, and the Old Partners program in Shanghai (21). Zhang and Yang compared different modes of community aging care services in China (22), and Chen and Han focused on the evolution of community aging care in Shanghai and put forward recommendations for China's growing aging care industry (23).

The above literature shows that family support and mutual help play an important role in aging care. In the context of aging, the increasing demand for care of the older adult and the decreasing time of young children form a prominent contradiction. Therefore, mutual aid for aging care has become the choice of more and more families. By forming a group of several families to help each other, on the one hand, senior citizens in a group can help each other as much as possible, and on the other hand, children can respond to common needs by taking turns on duty. In this case, how much time should be spent on mutual aid for the older adult in terms of time and how much influence the size of the group has in terms of space are two very important questions when studying mutual aid for aging care. The above two questions are answered by building a model in this article.

## Models

In the process of building an overlapping generations model, it should be noted that these models often have similar structures from the perspective of appearance; that is, these models are set separately from the perspectives of representative agents, representative enterprises, and governments, and then by setting exogenous variables such as time preference, endowment insurance contribution rate, and total factor productivity, the value of the core variable can be solved, such as Yew and Zhang (24), Miyazaki (25), and Yan (26). This structural similarity is characteristic of the overlapping generations model. However, when studying different economic problems, it is necessary to make the content of the model different according to the different research objects or influencing factors. For example, when studying the problem of delayed retirement, it is necessary to consider the income of representative agents after delayed retirement and the changes in the income and expenditure of pension insurance funds faced by the government in the model.

The most basic overlapping generation model describes such an economic phenomenon. The utility of a simple representative agent only considers consumption in the young and gerontic periods, and a representative agent works at a young age to earn income and provide gerontic consumption through savings. Representative agents can determine the duration of work and the amount of savings, which determine the cost of the enterprise, namely the amount of capital and labor. In this scenario, representative agents and enterprises adjust their behavior through the invisible hand of the market in order to maximize their own interests. Representative agents choose the time they work and the amount of savings they make, while representative enterprises choose the amount of labor and capital they use. Representative agents will certainly increase savings and working hours under high interest rates and high wages, but representative enterprises will reduce demand because of the high cost of using capital and labor, which will lead to representative agents reducing their requirements for interest rates and wages. Finally, market equilibrium is realized.

In this article, the overlapping generation model is used to study the problem of mutual aid for aging care, and factors such as the time to participate in the group, the utility of parents with different personalities, and the working time should be considered. Therefore, the logical flow for building the model is as follows: First, representative agents, representative enterprises, and government departments are considered in the model. The problem that representative agents consider is how to maximize their utility, which is related to their consumption in youth, their consumption in old age, and their parents' welfare. Representative agents can choose how much they save, how long they care for their parents, and how long they work to maximize their utility. Second, representative enterprises pay attention to their profits. They maximize their profits by selecting the amount of labor and capital used. Third, the government is responsible for the balance of income and expenditures in the endowment insurance fund. Providing a higher pension can increase the welfare of the representative agent, but it will also cause a gap in the fund, so this also forms a constraint. Fourth, after the relevant parameters are set, to achieve macroeconomic equilibrium, the value of the time to participate in the mutual aid group can be calculated under the constraints of labor market equilibrium, capital market equilibrium, utility maximization, enterprise profit maximization, and government pension fund balance. This value is the theoretical optimal value under the above conditions, which can provide theoretical guidance for mutual aid in aging care. Fifth, by changing the parameters in the model, we can further analyze the impact of these parameters on the optimal time, such as the size of a care group and the parents' personality preferences.

In view of the above considerations, this article constructs an overlapping generations model that includes representative agents, representative enterprises, the endowment insurance system constructed by the government, and the behavior of representative agents in mutual aid for aging care under the background of active aging. In the overlapping generation model to be constructed below, the influence of the time of participation in mutual aid and the size of the mutual aid group is analyzed by considering the behavior changes after participating in the mutual aid group of the representative agents.

### Representative agents

The representative agents experience two periods of youth and old age. In youth, agents spend a certain amount of time working to obtain income for consumption and saving. Working time is affected by caring for the older adult. The more time young people spend caring for their parents, the less time they are able to work. In old age, agents consume their savings from their youth and receive their old-age pensions. Under the gradually grave background of the problem of an aging population, more and more families begin to seek mutual aid with the increase in dependency ratio, that is, the children of several families gather older adults of each family together and take turns to provide care. Taking the mutual aid model in Shaanxi Province described in the “Introduction” section as an example, this article considers the behavior of representative agents taking care of older adults, in turn, when they are young. In this way, these families can help each other. On the one hand, the older adult can get better care. On the other hand, the younger generation can also take turns to satisfy the common requirements of older adults from different families because of mutual aid, thus reducing the total time for aging care. In addition, taking care of parents can take up both work time and leisure time, and mutual aid for aging care often has a certain institutional nature, which inevitably takes up work time. Individuals cannot earn income through work during their leisure and care time, so there is no economic difference between the two. Considering that this article analyzes from the perspective of economic utility, the care time studied in this article is the part that occupies work time and thus affects personal economic utility. According to the above conditions, the budget constraint equations of representative agents in youth and old age are obtained. The consumption *C*_1, *t*_ of the young generation at time *t* is subject to the following constraints:


(1) $$\begin{array}{l} C_{1,t}=\left(1-\left(1+\rho_{t}\right)\cdot \theta +\varphi \cdot \rho_{t}\cdot \theta \right)\cdot w_{t}\cdot \left(1-\tau \right)-s_{t} \end{array}$$

where θ is the time that representative agents only take care of their own parents. In this case, the working time is 1−θ, ρ_*t*_ is the extra time required to pay after participating in mutual aid, which is a certain proportion of θ, φ is the size of a mutual aid group for aging care. After participating in mutual aid projects, the time spent by agents to care for older adults is the sum of θ and ρ_*t*_·θ, that is, (1+ρ_*t*_)·θ. However, since some common requirements can be met by caring for older adults, in turn, under the mode of mutual aid for aging care, representative agents can gain φ·ρ_*t*_·θ time by participating in a mutual aid group of φ families. Finally, the working time of representative agents is 1−(1+ρ_*t*_)·θ+φ·ρ_*t*_·θ. *w*_*t*_ is the salary of the younger generation at time *t*, τ is the contribution rate of pension insurance, and *s*_*t*_ is the savings of the younger generation at time *t*. Equation (1) shows the consumption of representative agents in their youth. In this formula, the benchmark working time minus the time to care for parents is the actual working time. The actual working time multiplied by the wage per unit time *w*_*t*_ is the wage, but the agents' disposable income needs to be paid for the endowment insurance at the ratio of τ. Finally, disposable income is divided into two parts, one is for saving for the gerontic, and the rest is for consumption.

The younger generation at time *t* is the older generation at time *t*+1, their consumption *C*_2, *t*+1_ in old age is subject to the following constraints:


(2) $$\begin{array}{l} C_{2,t+1}=s_{t}\cdot R_{t+1}+I_{t} \end{array}$$

where *R*_*t*+1_ = 1+*r*_*t*+1_, *r*_*t*+1_ is the interest rate of savings, and *I*_*t*_ is the pension received from the government at time *t*. Equation (2) shows the consumption of representative agents in their old age. The consumption in old age comes partly from savings in young age and partly from the pension.

According to the basic idea of the overlapping generations model, the representative agents' utility is affected by the consumption *C*_1, *t*_ in their youth and the consumption *C*_2, *t*+1_ in their old age. Moreover, since the number of children and human capital are introduced into the utility function when studying the birth rate and education, this article draws lessons from these literatures, and the utility of parents *P* is introduced into the utility function when this article studies the issue of parents' old-age care. The utility of representative agents can be expressed by the following utility function:


(3) $$\begin{array}{l} U=\ln\;\left(C_{1,t}\right)+\beta \cdot \ln\;\left(C_{2,t+1}\right)+\gamma \cdot \ln\;\left(P\right) \end{array}$$

where β is the discount rate of time, γ is the representative agents' preference for parents, and *P* is the utility of parents. According to the APIM model used by Kenny and Cook (27) and Rippon et al. (28), when parents are dominated by partner effects, they prefer to wait with their children, while when they are dominated by actor effects, they prefer to stay with more people. The utility of parents can also be written in Cobb-Douglas form:


$$\begin{array}{l} P=\varphi^{\lambda }\cdot {\left[\left(1-\rho \cdot \varphi \right)\theta +\rho \cdot \theta \right]}^{1-\lambda } \end{array}$$

where λ and 1–λ are parents' psychological preferences. The larger the value of λ is, parents are more affected by the actor effect, which means that the more people there are, the more satisfied parents will be. In this article, the larger the size φ of a mutual aid group, the more satisfied the parents will be. The smaller the value of λ, that is, the larger the value of 1–λ, indicating that parents are more affected by the partnership effect, which means that parents are more willing to get along with their children. Equation (3) shows the utility of representative agents. They pay attention to their consumption in their youth, their consumption in old age, and their parents' utility. Changing the amount of savings and the time to care for their parents can change their utility. To calculate the optimal proportion of time, the value of the proportion of time is analyzed under some constraints to achieve the maximum utility.

The optimal choice faced by representative agents is to maximize utility under the constraints of Equations (1) and (2):


$$\begin{array}{l} \max U=\max\left[\ln\;\left(C_{1,t}\right)+\beta \cdot \ln\;\left(C_{2,t+1}\right)+\gamma \cdot \ln\;\left(P\right)\right] \end{array}$$

By solving the personal optimal utility Equation (3) under the constraints of Equations (1) and (2), the partial derivatives of ρ_*t*_ and *s*_*t*_ are obtained. Under optimal conditions:


(4) $$\begin{array}{l} \frac{\left(\varphi -1\right)\theta w_{t}\left(1-\tau \right)}{C_{1,t}}+\gamma \frac{\left(1-\varphi \right)\theta \left(1-\lambda \right)}{P}=0 \end{array}$$

(5) $$\begin{array}{l} -\frac{1}{C_{1,t}}+\beta \frac{R_{t+1}}{C_{2,t+1}}=0 \end{array}$$

Then, optimal personal savings *s*_*t*_ and optimal time ratio ρ_*t*_ for mutual aid are calculated by simultaneously solving Equations (4) and (5):


(6) $$\begin{array}{l} \rho_{t}\;=\frac{s_{t}\left(\gamma -\gamma \lambda \right)\;+\;w_{t}\left[-\theta +\gamma \left(-1\;+\;0\right)\left(-1\;+\;\lambda \right)\right]\left(-1\;+\;\tau \right)}{w_{t}\left[-1\;+\;\gamma \left(-1\;+\;\lambda \right)\right]\left(-1\;+\;\tau \right)\left(-1\;+\;\varphi \right)}\; \end{array}$$

(7) $$\begin{array}{l} s_{t}=\frac{I_{t}+R_{t+1}w_{t}\beta \left(-1+\tau \right)\left(1+\theta \left(-1+\rho_{t}\left(-1+\varphi \right)\right)\right)}{R_{t+1}\left(1+\beta \right)} \end{array}$$

### Representative enterprises

According to the method of analyzing the representative enterprise based on the overlapping generations model, this article sets that the enterprise has a C-D production function and that the output of the enterprise *Y*_*t*_ is determined by capital and labor. The specific production function is as follows:


(8) $$\begin{array}{l} Y_{t}=AK_{t}^{\phi }{\left[\left(1-\left(1+\rho_{t}\right)\cdot \theta +\varphi \cdot \rho_{t}\cdot \theta \right)L_{t}\right]}^{1-\phi } \end{array}$$

where *Y*_*t*_ is the output level of the economy, *A* is the total factor productivity, representing the level of technological progress in the economy, *K*_*t*_ is the capital stock in the economy, and the last part is the product of the number of young people and working hours, representing the quantity of labor in the economy. and 1−ϕ are the share of income from capital and labor, respectively. Given the interest rate and wages, enterprises can choose the quantity of labor and capital to maximize their profits. Formula (8) shows the output of the enterprise. The output depends on the input of capital stock and effective labor. When considering the problem of mutual aid for aging care, effective labor according to working hours is used, and other parts are set according to the traditional model.

According to the setting method of capital depreciation by Fanti and Gori (29) and Li and Lin (30), the depreciation rate is set as 1 in this article, and the profit π_*t*_ of the enterprise is


(9) $$\begin{array}{l} \pi_{t}\text{=}AK_{t}^{\phi }{\left[\left(1-\left(1+\rho_{t}\right)\cdot \theta +\varphi \cdot \rho_{t}\cdot \theta \right)L_{t}\right]}^{1-\phi }\\ -R_{t+1}\cdot K_{t}-w_{t}\left(1-\left(1+\rho_{t}\right)\cdot \theta +\varphi \cdot \rho_{t}\cdot \theta \right)L_{t} \end{array}$$

Equation (9) shows the profit of the enterprise. The first part on the right side of the equation is the output, and the two parts subtracted are the cost of capital (interest) and the cost of labor (wages). According to Equation (9), the problem faced by enterprises is also transformed into how to maximize profits by adjusting the amount of labor and capital used.

The output of per labor *y*_*t*_ and the capital of per labor *k*_*t*_ can be calculated by dividing both sides of Equation (9) by the effective labor (1−(1+ρ_*t*_)·θ+φ·ρ_*t*_·θ)*L*_*t*_, and equation (8) of the production function can be written as $y_{t}=Ak_{t}^{\phi }$.

By solving the problem of maximizing the profit, the following equation is obtained:


(10) $$\begin{array}{l} R_{t+1}=A\cdot \phi \cdot k_{t}^{\phi -1} \end{array}$$

(11) $$\begin{array}{l} w_{t}=A\left(1-\phi \right)k_{t}^{\phi } \end{array}$$

### Endowment insurance system

The government is responsible for maintaining the balance of income and expenditure in the endowment insurance funds. Referring to the model of Miyazaki (25), the pay-as-you-go endowment insurance model is constructed, and the endowment insurance fund paid by the younger generation is allocated to retired older workers. To maintain the balance of income and expenditure of the endowment insurance fund, the following constraints need to be met:


(12) $$\begin{array}{l} L_{t}\cdot I_{t}=\left(1-\left(1+\rho_{t+1}\right)\cdot \theta +\varphi \cdot \rho_{t+1}\cdot \theta \right)\cdot w_{t+1}\cdot \tau \cdot L_{t+1} \end{array}$$

where *L*_*t*_ is the number of young people at time *t*, and the left side of Equation (12) represents the pension received by the young representative agent after entering old age. The right side of Equation (12) represents the total pension insurance paid by the younger generation at time *t*+1. Under the pay-as-you-go system, the left and right sides of Equation (12) are equal. Equation (12) shows the constraints faced by the government, that is, the balance of income and expenditure of the pension insurance fund. To achieve the balance of pension insurance funds, the contribution rate of pension insurance and the level of insurance payment should be restricted. The following equation is obtained by dividing both sides by *L*_*t*_:


(13) $$\begin{array}{l} I_{t}=\left(1-\left(1+\rho_{t+1}\right)\cdot \theta +\varphi \cdot \rho_{t+1}\cdot \theta \right)\cdot w_{t+1}\cdot \tau \cdot n_{t} \end{array}$$

where *n*_*t*_ is the fertility rate, that is, *n*_*t*_ = *L*_*t*+1_/*L*_*t*_. In Equation (13), the constraints faced by the government are expressed in per capita form by introducing the birth rate, but the significance of this equation is still that the government should achieve the balance of income and expenditure on pension insurance by changing the contribution rate and payment level.

### Macroeconomic equilibrium

In this model, the macroeconomic equilibrium means that given the initial capital *K*_0_, the representative agent in the economy chooses variables {*C*_1, *t*_, *C*_2, *t*+1_, ρ_*t*_, *s*_*t*_}, so that the capital and labor {*K*_*t*_, (1−(1+ρ_*t*+1_)·θ+φ·ρ_*t*+1_·θ)*L*_*t*_}, the prices of production factors {*r*_*t*_, *w*_*t*_}, and pension insurance {τ, *I*_*t*_} meet the following conditions:

1. Under the conditions that the prices of capital and labor {*r*_*t*_, *w*_*t*_} and the policies of pension insurance {τ, *I*_*t*_} are determined, representative agents maximize their own welfare by choosing their consumption in two periods, savings in their youth, and time to participate in a mutual aid group.
2. Under the premise that the prices of production factors {*r*_*t*_, *w*_*t*_} are given, representative enterprises maximize their profits by selecting the amount of capital and labor.
3. The equilibrium of the labor market is realized, that is, the labor supply of young people equals the demand of enterprises for labor.
4. The equilibrium of the capital market is realized. The supply of capital in the economy is equal to the demand for capital, that is, the total amount of savings is equal to the total amount of capital, that is:

(14) $$\begin{array}{l} K_{t+1}=s_{t}\cdot L_{t} \end{array}$$

Both sides of Equation (14) are divided by effective labor, and the dynamic accumulation equation of per capita capital can be obtained as follows:

(15) $$\begin{array}{l} K_{t+1}=\frac{K_{t+1}}{\left(1-\left(1\;+\;\rho_{t+1}\right)\cdot \;\theta \;+\varphi \;\cdot \;\rho_{t+1}\cdot \;\theta \right)L_{t+1}}\;=\\ \frac{s_{t}}{\left(1-\left(1\;+\;\rho_{t+1}\right)\cdot \;\theta \;+\varphi \;\cdot \;\rho_{t+1}\cdot \;\theta \right)n_{t}} \end{array}$$

5. The income and expenditures of the endowment insurance fund are balanced. The income of the endowment insurance fund is equal to the expenditure of the endowment insurance fund; that is, Equations (12) and (13) are satisfied.

According to these constraints set in this section, representative agents can choose the time to participate in a mutual aid group and save in their youth to maximize the utility. However, the price of labor and the income and expenditure of endowment insurance need to be given. Similarly, after the capital price and labor price are given, representative enterprises can also maximize profits by selecting the amount of labor and capital used. Based on the equilibrium of the capital market and labor market, the interest rate and wage level that can realize market clearing are obtained. Interest rates and wages, in turn, provide constraints for representative agents and enterprise decision-making. Therefore, the equilibrium analysis has also become the key to solving the overlapping generation model.

### General-equilibrium analysis

The production function in this article is assumed to be a neoclassical production function, without considering exogenous technological progress. When the economy converges to the steady state, the equations $k_{t+1}=k_{t}=k^{*}$ and $n_{t+1}=n_{t}=n^{*}$ are satisfied.

Combining the above conditions, the following equation can be obtained by substituting Equations (6)–(13) into Equation (15):


(16) $$\begin{array}{l} k^{*}=\frac{n^{*}\left[\phi -\tau \left(\phi -1\right)\right]}{A\cdot \beta \cdot \phi \left(\phi -1\right)\left(\tau -1\right)} \end{array}$$

According to the equilibrium state calculated by Equation (16), comparative static analysis can be performed. Equation (16) shows the capital level under the equilibrium state. This result represents the equilibrium of the capital market and the labor market. After these are given, the optimal values of the representative agent utility maximization and the enterprise profit maximization can be calculated.

## Parameters setting and numerical simulation

### Parameters setting

According to the existing literature and the actual situation in China, the relevant parameters in the above model are set. In terms of setting two periods of 30 years, the reasons are as follows: Based on the Statistical Bulletin of China's Health Development in 2021 issued by the National Health and Health Commission, the average life expectancy of Chinese residents reached 78.2 years in 2021, an increase of 0.27 years compared with 77.93 years in 2020. Therefore, this article assumes that the representative agents will live to 80 years old and receive education before the age of 20 years, and then experience 30 years of youth and 30 years of old age, consistent with the setting of Yan (26). The setting of other parameters and the reasons are as follows:

1. Time for representative agents to take care of their parents. By mutual aid, representative agents can form a group to take care of each other's parents and reduce the total time spent on care. The benchmark care time θ in this article is the time for representative agents to take care of their parents without mutual aid. Referring to the results of Jing et al. (31), the benchmark care time θ is set to 0.38. Considering the importance of the parameter θ, the sensitivity analysis is carried out later.
2. Contribution rate of pension insurance τ. According to the provisions of the No. 38 Document in 2005 and the No. 13 Document in 2019 issued by the State Council, employees pay endowment insurance at 8% of their wages and deposit it into their own accounts, and the enterprise pays the endowment insurance at 16% of the employee's wages and deposits it in the social pooling account. Since the part of the personal account will only be distributed to the individual, the personal account is equivalent to the savings in this article, and only the part paid by the enterprise is the pension insurance named pay-as-you-go pension insurance. In view of this, the contribution rate for pension insurance τ is set at 16%.
3. Discount rate of time β. The discount rate of time β reflects the utility level of representative agents' intertemporal consumption discounted to the current period in the model. The smaller this parameter is, the more attention is paid to consumption at a young age. According to Pecchenino and Pollard (32), the discount rate is 0.98 per year. The two periods of the model built in this article are 30 years each, so the discount rate of time in this article is 0.99^30^≈0.74.
4. Representative agents' preference for parents γ. γ indicates the degree of emphasis on parents and themselves. According to Yan (26), children's attention to their parents' utility is similar to that of their own utility. Therefore, this article assumes that individuals have the same degree of preference between themselves and their parents, that is, γ = 1.
5. Total factor productivity *A*. Different experts and scholars have different estimates of China's total factor productivity. For example, Wang calculated China's total factor productivity as 7.47 based on China's 30 years of historical experience data (33), Lu and Lian estimated that the total factor productivity of China's provinces is between 3.56 and 4.68 (34), Kang and Chu calculated that the result is 2.05 (35). The average of the highest and lowest values is set as the value of total factor productivity, that is, *A* = 4.8. In addition, through sensitivity analysis on the value of this parameter, its value does not affect the value of the core variable.
6. Share of capital ϕ and share of labor 1−ϕ. Guo and Ren (36) and Liu et al. (37) calculated the share of capital and labor in China, and the results show that the share of capital in China is basically 0.3–0.5. Therefore, 0.4 is taken as the share of capital in this article, and the share of labor is 0.6.
7. Fertility rate *n*. According to the data of the China Statistical Yearbook, the fertility rate has dropped below 1% since 2020. According to the fertility rate in the 2021 National Economic Statistics Bulletin issued by the National Bureau of Statistics, the annual fertility rate is set at 7.52‰, so *n* = (1+0.00752)^30^ = 1.25.
8. Size of group φ. The parameter φ is a key point of this study. φ is a positive integer and φ>1, that is, at least two or more families form a mutual aid group for aging care. Moreover, limited by time and space constraints such as the number of acquaintances, the number of nearby residents, energy, and ability, the size of the group cannot be unlimited. In view of this, the benchmark size of the group is selected as 5, and the sensitivity of this parameter is analyzed later.
9. Parents' psychological preference λ. Parents' psychological preferences reflect their characters. Outgoing parents prefer to communicate with more people, so they prefer a larger group size. Some parents prefer to get along with their children, so they prefer their children's company. Different parents have different psychological preferences. The parents whose preference λ is equal to 0.5 are defined as the neutral group, and the two motivations of the neutral group are the same and can be replaced by each other. The parents whose preference λ is < 0.5 prefer to stay at home with acquaintances, which are called introverted parents, while the parents whose preference λ is >0.5 prefer to communicate with more people and participate in the mutual aid group, which are called extroverted parents. The neutral group is taken as the benchmark for analysis in this article. In addition, the effects of different preferences on the welfare of the older adult and the optimal time for children to participate in the mutual aid group are also analyzed.

### Numerical simulation results

By substituting the benchmark values of the parameters into the model, the optimal time for representative agents to participate in the mutual aid group is calculated. In the base case, the optimal proportion of time is 9.31%, which means representative agents need to spend 0.035 time in the mutual aid group when the benchmark care time θ is set to 0.38. In other words, this also means that representative agents' parents can enjoy the care provided by the other four members, each of whom provides 0.035 time. From the perspective of working hours, if the representative agents only take care of their own parents, the working time 1−θ is 0.62 in the base case. After participating in the mutual aid group, the working time of representative agents is 0.76, increased by 0.14. The annual interest rate in the overlapping generations model is 3.30%.

## Sensitivity analysis

In the study of mutual aid for aging care, two of the most important parameters are the time for representative agents to take care of their parents θ and the size of the group φ. These two factors affect the optimal proportion of time ρ through the benchmark care time θ in the time dimension and the size of the group φ in the space dimension. Therefore, the impact of these two factors on the optimal proportion of time ρ is analyzed as follows.

### Sensitivity of the time for representative agents to take care of their parents θ

**Figure 1:**
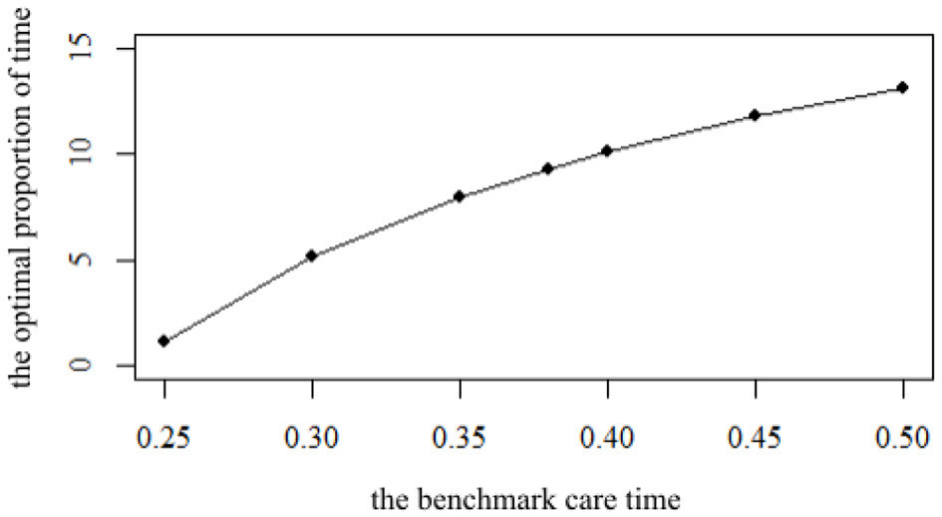
The impact of the benchmark time θ on the optimal proportion of time ρ.

Based on the principle of “everyone for me, I for everyone”, participating in a mutual aid group can reduce the time for representative agents to take care of their parents. The benchmark care time θ is set to 0.38 in the parameter setting above, and the optimal proportion of time ρ is calculated as 9.31%. Considering that the time for representative agents to take care of their parents may be different, the sensitivity of the impact of θ on ρ is analyzed. The impact of different benchmark care time θ for representative agents to take care of their parents θ on ρ is shown in Figure 1.

It can be seen from Figure 1 that with the increase of θ, the proportion increases correspondingly, but the increase is decreasing. When the time for representative agents to take care of their parents θ is 0.2, the optimal proportion of time ρ is only 1.15%. When θ is 0.25, the optimal proportion of time increases to 5.13%, an increase of 3.98%. When θ increases from 0.45 to 0.5, the optimal proportion of time ρ increases from 11.75 to 13.08%, an increase of 1.33%. The growth rate obviously shows a decreasing trend. It can be found that the longer the benchmark time for representative agents to take care of their parents, the heavier their burden of taking care of their parents is. Therefore, these representative agents will also participate more actively in the mutual aid group to obtain more returns. However, with the increase of θ, the time to participate in the mutual aid group increases, but the marginal impact is reduced.

### Sensitivity of the size of group φ

**Figure 2:**
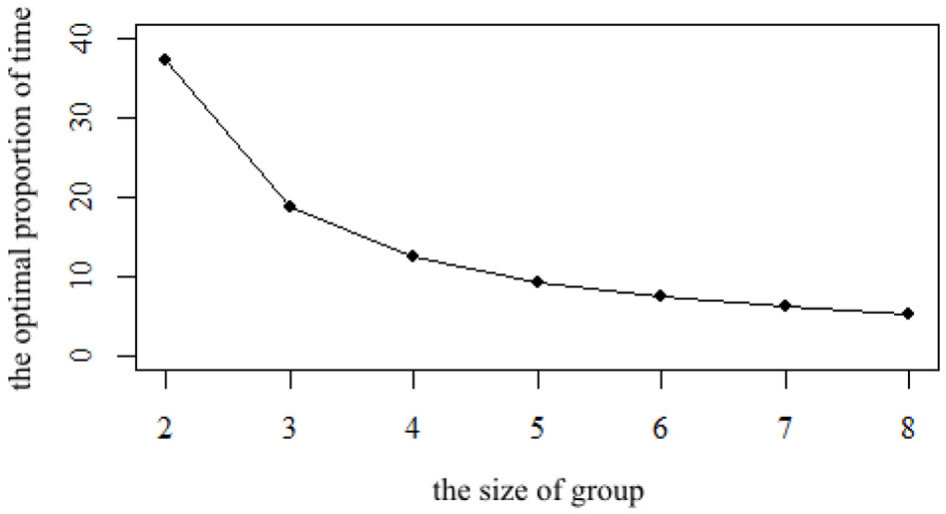
The impact of different size φ on the optimal proportion of time ρ.

The size of the group is also an important factor. Since a group needs at least two families to form, the minimum size of the group φ is set at 2. Due to the limitations of communication scope, space distance, and personal ability, it is not practical to set up a very large team, and the largest size is set at 8 in the sensitivity analysis. The impact of different size φ on ρ is shown in Figure 2.

It can be seen from Figure 2 that with the increase of φ, the proportion ρ decreases. If there are only two families in the group, representative agents need to spend 37.25% of their benchmark care time θ to participate in the mutual aid group. If the size of the group φ increases to three families, the proportion decreases to 18.62%. With the increase in size, the proportion ρ continues to decline. When the team size reaches eight, the proportion ρ is only 5.32%, showing a significant downward trend. In terms of the downward trend, with the increase in the size of the group, the decline speed of ρ slows down. When the size of the group increases from 2 to 3, ρ decreases by 18.63%, whereas when the size of the group increases from 7 to 8, it only decreases by 0.89%, which means that the marginal impact of reducing ρ will be smaller when the size reaches a certain degree. Limited by the various factors mentioned above, the larger the group, the better. The benchmark size is set at five, and the size of the group [[Mathtype-mtef1-eqn-154.mtf]] only decreases from 9.31 to 7.45% if the size increases to six, a decrease of 1.86%. Considering that in this case, the impact of continuing to increase φ is relatively small, but there are many difficulties in increasing φ in reality, so the setting of parameter in this article is reasonable.

### The combined effect of θ and φ

**Figure 3:**
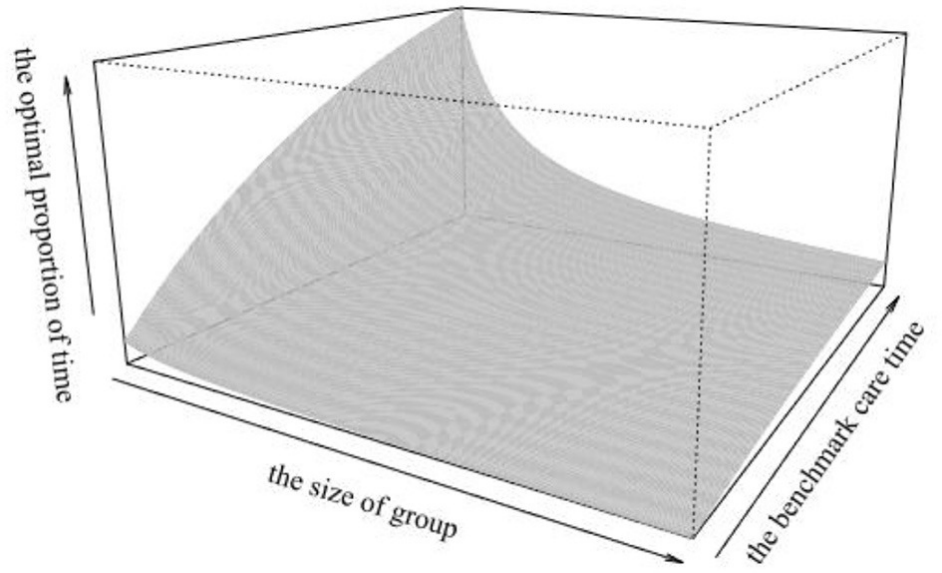
The impact of the benchmark time θ and the size φ.

To intuitively reflect the impact of θ and φ on the optimal proportion of time ρ, the optimal proportion of time ρ for representative agents to participate in a mutual aid group when the two parameters θ and φ are changed, as shown in Figure 3.

It can be seen from Figure 3 that the impact of θ and φ on the optimal proportion of time ρ is the same as the trend when only one parameter changes, that is, the longer the benchmark time θ, or the larger the size of group, the longer the representative agents choose to participate in the mutual aid group. In addition, increasing the benchmark care time θ in a small group or increasing the size of the group φ at a lower base time has a small impact on the optimal proportion of time ρ. Only when both and φ are at an appropriate level, the optimal proportion of time will be at a high level.

## Further analysis of parents with different preferences

**Figure 4:**
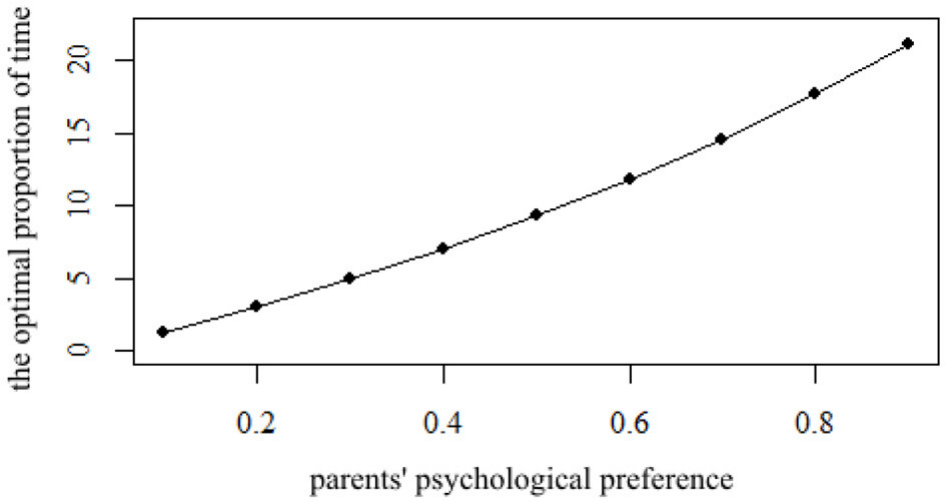
The impact of parents' psychological preference λ on the optimal proportion of time ρ.

In this article, the neutral group with λ = 0.5 is selected as the benchmark. According to the APIM model, the influence of the partnership effect and actor effect on the utility of such groups is not different, and these two effects can replace each other. However, there are differences in parents' preferences. Extroverted parents prefer to communicate with more people, so increasing the size of the group can increase its utility. Introverted parents are different from extroverted parents, and enjoying the care of their children can increase their utility. From the perspective of personal utility maximization, the optimal proportion of time ρ varies with parents' preferences. The change in optimal proportion of time ρ based on the maximization utility (parameter of parents' psychological preference λ increased from 0.1 to 0.9) is shown in Figure 4.

It can be seen from Figure 4 that with the increase in parents' psychological preference λ, the partner effect of parents becomes stronger, and the optimal proportion of time ρ selected by children based on the maximization of utility is longer. That is to say, the children of extroverted parents have a longer optimal proportion of time ρ to participate in a mutual aid group, which matches their intuitive expectations. This is because extroverted parents prefer to participate in a mutual aid group, and their children will also pay more attention to taking care of the older adult by relying on mutual aid groups. When parents' psychological preference λ is 0.1, they tend to let their children take care of them. In this case, to maximize utility, the optimal proportion of time ρ of children is 1.29%. This result shows that for extremely introverted parents, their children tend to choose less time for mutual aid groups. When parents' psychological preference λ is 0.9, parents like to form groups to enjoy aging care, and the optimal proportion of time ρ of children has increased to 21.12%, which means that extroverted parents' children will spend more time in mutual help groups. In terms of the degree of increase, with the parents' psychological preference λ getting bigger, the marginal added value of ρ is also increasing. When the parents' psychological preference λ increases from 0.1 to 0.2, the optimal proportion of time ρ of their children increases from 1.29 to 1.91%, an increase of 1.75%. When the parents' psychological preference λ increases from 0.8 to 0.9, the optimal proportion of time ρ increases from 17.68 to 21.12%, an increase of 3.44%. The comparison of the two results reflects that children of extroverted parents not only have a longer optimal proportion of time ρ, but also have a greater marginal impact.

**Figure 5:**
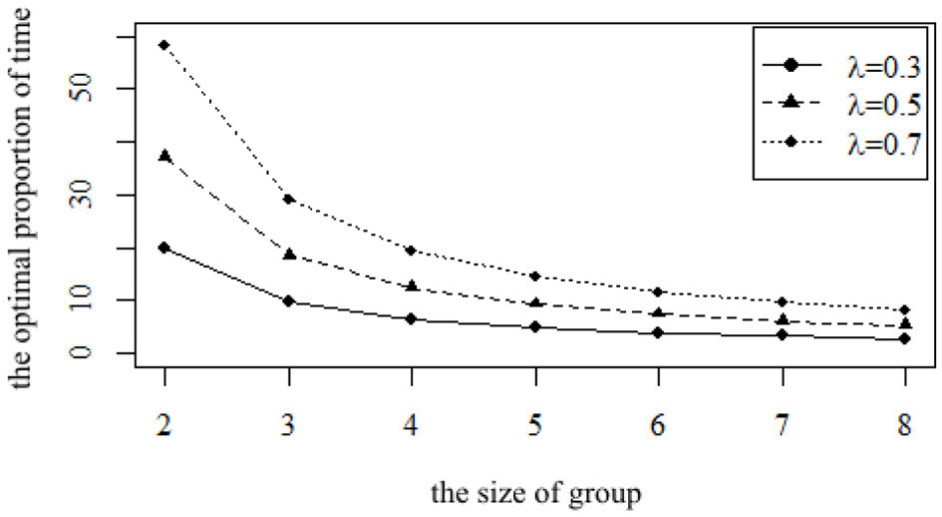
The impact of the size φ on the optimal proportion of time ρ for parents with different preferences.

For parents with different preferences, extroverted parents, neutral parents, and introverted parents have different preferences for the size of the group, so the children of these three types of parents will have different optimal proportions of time to participate in a mutual aid group. To reflect the influence of the size of the group with different preferences, 0.3, 0.5, and 0.7 are selected as representatives of introverted parents, neutral parents, and extroverted parents, respectively. The impact of size on the optimal proportion of time from the perspective of children and the impact on the utility of older adults from the perspective of parents are analyzed, respectively. The impact of different sizes on ρ for parents with different preferences is shown in Figure 5.

The three curves in Figure 5 show the optimal proportion of time ρ for children to participate in the mutual aid group when facing parents with different preferences. On the whole, whether introverted parents, neutral parents, or extroverted parents, with the increase in the size of the group, more children will participate in the group, and the optimal proportion of time ρ for children to participate in the mutual aid group will also decrease. Moreover, whether introverted parents, neutral parents, or extroverted parents, the marginal impact of increasing size on their children's optimal proportion of time ρ decreases. From the comparison of the three groups, the optimal proportion of time ρ for children with extroverted parents is higher than that of children with introverted parents and neutral parents. However, as the size of group increases, the difference in becomes smaller.

**Figure 6:**
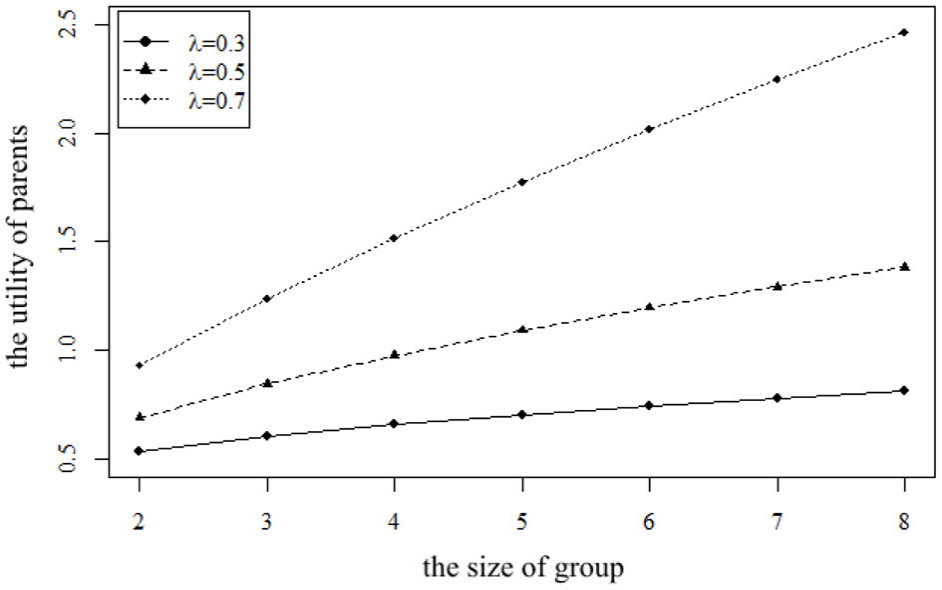
The impact of the size φ on the utility of parents *P* for parents with different preferences.

From the perspective of parents' utility in their old age *P*, the size of the group also has different effects on the utility of parents with different preferences. The impact of different sizes on *P* for parents with different preferences is shown in Figure 6.

It can be seen from Figure 6 that no matter what kind of parents they are, their utility increases with the increase in group size. This is because the increase in size means that parents can enjoy more care. However, due to the different preferences of parents, they have different utilities through the mutual aid group. The mutual aid group naturally has a communication function, so compared with neutral parents and introverted parents, extroverted parents can get more benefits from the mutual aid group. When the size of the group is small, the gap between the utility of extroverted parents and the other two groups is small. With the increase in the size of the group, extroverted parents get more satisfaction, so the gap expands.

## Conclusion and suggestions

Through the above analysis, the following main conclusions are drawn. First, for children who usually take care of their parents for a long benchmark time, the optimal time to participate in the mutual aid group based on personal utility maximization is also long. However, with the increase in benchmark time, the added value decreases. Second, as the size of the group increases, the time for representative agents to participate in the mutual aid group gradually decreases. When the size of the group is small, increasing the size of the group can quickly reduce the time for children to participate in the mutual aid group. However, when the group size reaches a certain level, the impact of continuing to increase the size becomes smaller. The impact of group size shows that, if possible, forming a larger group will be more conducive to reducing the care time of children. From the actual situation in Shaanxi Province, China, if the size of most mutual aid groups at this stage only includes 2–3 families, there is no doubt that the group size has the motivation to expand. Third, the analysis of parents with different preferences shows that the more extroverted parents are, the longer their children participate in the mutual aid group. This result shows that the model of mutual aid for aging care is highly correlated with parents' personalities. Since mutual aid has a natural social attribute, this model is more friendly to people who like to socialize. On the one hand, this result explains the importance of letting the older adult participate in social activities, and on the other hand, it also explains why the mutual aid group should be composed of relatives, friends, and neighbors. Fourth, for children of parents with different personalities, the children of extroverted parents participate in the mutual aid group for a longer time than the children of introverted parents and neutral parents. For parents with different personalities, extroverted parents get more benefits from mutual aid for aging care, while neutral parents and introverted parents get fewer benefits from mutual aid for aging care. This result shows that when children contribute the same amount of time to mutual support, the benefits of parents with different personalities are different. To increase benefits, children have the motivation to encourage their parents to engage in more social activities, which can make parents feel happy more easily.

Based on the conclusions, the following suggestions are proposed. First, the government should provide convenience in place, time, and other aspects of the mutual aid for aging care and promote more families to participate in the mutual aid group. At present, older adults mainly rent a house or use the house of a group member as a place for mutual support in China, which limits the motivation for mutual aid. If the government can provide a place, it will certainly promote more people to participate in mutual aid. In rural China, as young people enter the cities, there are many idle houses. If the government can rent these idle houses to provide a place for the older adult, which can reduce the government's expenditure on building nursing homes, it is undoubtedly beneficial to all parties. In addition, compared with the spontaneous formation of a group by the older adult, it is easier to expand the group size if the government provides a place for mutual aid, and a larger group size means that children need to pay less time.

Second, the community should increase senior citizen sports centers, encourage more older people to participate in social activities, eliminate social fears of introverted older people, and increase the welfare of older people through mutual aid. Because extroverted parents are more able to benefit from mutual aid, providing community activities to promote communication with others will increase the benefits obtained without increasing input by improving personality. In Singapore, the government has extensively carried out the “Leling Movement” and established Leling clubs in every community. The older adult can enjoy health examinations, birthday celebrations, tourism, and other services in the club. The club has a ceramic art room, a martial arts hall, a wine mixing room, a fitness room, a band room, a painting room, and so on. There are fixed areas for all kinds of activities. Every older person who comes to the community to participate in activities can make friends according to their hobbies. Learning from relevant practices and enhancing the enthusiasm of the older adult to participate in activities will not only directly promote their happiness, but also indirectly increase the benefits of mutual aid for aging care.

Third, children who are responsible for supporting their parents should be encouraged to participate more actively in the mutual aid group. The mutual aid awareness of “everyone for me, I for everyone” should be enhanced so that children and parents can benefit from actively participating in mutual aid. The enthusiasm of individuals to participate in mutual aid groups is affected by many factors. Being able to realize that participating in mutual aid can increase the benefits of individuals and families is the basis, and enhancing the sense of responsibility provides motivation for participating in mutual aid. The sense of responsibility can come from voluntary or from relevant regulations. The model of “Time Bank” in Switzerland provides a good reference. Young people can deposit their time in the time bank by participating in voluntary service and getting a period of service in their old age. In this way, to meet the needs of their old age, the responsibility of young people is enhanced. Referring to the model of the “Time Bank”, improving the management of the time bank and scientifically calculating the conversion amount of time are of great significance for the development of mutual aid for aging care.

This article analyzes the optimal proportion of time for children to participate in mutual aid and the sensitivity analysis of the benchmark care time and the size of the group is carried out. It is positive to solve these key problems in mutual aid for aging care, and it is also helpful to solve the aging problem in China. However, this article does not consider the differences between the care provided by their children and the care provided by other members of the group, and this article does not limit the transfer proportion of children's responsibility, which is awaiting further study.

## Data availability statement

The original contributions presented in the study are included in the article/supplementary material, further inquiries can be directed to the corresponding author.

## Author contributions

All authors have made a substantial contribution to the conception and design, or analysis and interpretation of data, and the drafting and approval of this article.

## Footnotes

^1^The data are sourced from the *2021 National Bulletin on the Development of Aging Care*. Available online at: http://www.nhc.gov.cn/lljks/s10742/202210/e09f046ab8f14967b19c3cb5c1d934b5.shtml.

^2^The data were sourced from relevant news reports on the Xinhua Network.
